# An association between human hippocampal volume and topographical memory in healthy young adults

**DOI:** 10.3389/fnhum.2012.00338

**Published:** 2012-12-31

**Authors:** Tom Hartley, Rachel Harlow

**Affiliations:** Department of Psychology and York Neuroimaging Centre, University of YorkYork, UK

**Keywords:** voxel-based morphometry, spatial memory, gray matter, insula, temporal lobe

## Abstract

The association between human hippocampal structure and topographical memory was investigated in healthy adults (*N* = 30). Structural MR images were acquired, and voxel-based morphometry (VBM) was used to estimate local gray matter volume throughout the brain. A complementary automated mesh-based segmentation approach was used to independently isolate and measure specified structures including the hippocampus. Topographical memory was assessed using a version of the Four Mountains Task, a short test designed to target hippocampal spatial function. Each item requires subjects to briefly study a landscape scene before recognizing the depicted place from a novel viewpoint and under altered non-spatial conditions when presented amongst similar alternative scenes. Positive correlations between topographical memory performance and hippocampal volume were observed in both VBM and segmentation-based analyses. Score on the topographical memory task was also correlated with the volume of some subcortical structures, extra-hippocampal gray matter, and total brain volume, with the most robust and extensive covariation seen in circumscribed neocortical regions in the insula and anterior temporal lobes. Taken together with earlier findings, the results suggest that global variations in brain morphology affect the volume of the hippocampus and its specific contribution to topographical memory. We speculate that behavioral variation might arise directly through the impact of resource constraints on spatial representations in the hippocampal formation and its inputs, and perhaps indirectly through an increased reliance on non-allocentric strategies.

## Introduction

Recent evidence indicates that variations in the structure of the hippocampus are associated with individual differences in spatial behavior (Maguire et al., 2006). Structural change can take place after prolonged training in experts which may account for the observed associations (Woollett and Maguire, 2011), but hippocampal structure is also subject to experience-independent (e.g., genetic) influences (Stein et al., 2012) suggesting that some of the individual variation underlying performance differences may not depend on specific expertise or training. If this were the case, we might expect the association between hippocampal structure and spatial function to extend to the general population. The current study investigates the biological basis of individual differences in healthy young adults by analyzing volumetric measures from structural MRI against performance on a task expressly designed to tax hippocampal-dependent aspects of spatial representation and processing. Below, we outline key evidence and theoretical perspectives on the role of the hippocampus in spatial representation and behavior. We then briefly review previous work investigating links between brain structure and behavior in this domain, before outlining the basis for the task used in the present investigation.

A large body neuropsychological evidence indicates that the hippocampus is critical for the formation of new memories of personally experienced events (see e.g., Spiers et al., 2001; Ranganath, 2010) but it is also believed to play an important role in spatial memory and navigation (O'Keefe and Nadel, 1978). Neurophysiological evidence indicates that cells in the hippocampus and its neocortical inputs provide an allocentric representation of space (see Doeller et al., 2012 for a recent review). Specifically, neurons in the hippocampal formation of the rat (Place Cells, Grid Cells, Head Direction Cells, and Boundary Cells) have spatial firing fields (encoding location and heading) which are anchored with respect to the environment and largely independent of egocentric information (O'Keefe, 1976; Taube et al., 1990; Hafting et al., 2005; Solstad et al., 2008; Lever et al., 2009).

One suggestion is that the episodic and spatial functions of the hippocampus are linked by a common requirement to represent spatial information in an allocentric form (Burgess, 2002). In the context of navigation, allocentric representations would allow for efficient calculation of novel shortcuts and, more generally, would permit flexible spatial reasoning about locations beyond the immediate scope of perception (Tolman, 1948; O'Keefe and Nadel, 1978). In episodic memory, an allocentric representation could provide a common spatial reference frame or “cognitive map” for encoding the context in which events occur. In each case, allocentric representation is useful for the efficient encoding and flexible retrieval of information about the spatial relations of events and locations (Cohen and Eichenbaum, 1993).

More recent work has extended these ideas about hippocampal function, building on observations of a common “core” network of brain regions, centered on the hippocampus, and active during a range of tasks involving shifts of perspective. Both “self-projection” (Buckner and Carroll, 2007) and the more explicitly spatial “scene-construction” (Hassabis and Maguire, 2007) accounts suggest this network is involved in the common process of imagining and manipulating spatial scenes whether in episodic recall, imagination, future thinking, or navigation. An implemented model of the mental manipulation of spatial scenes (Byrne et al., 2007; see Bird and Burgess, 2008, for review) suggests that the ability to imagine the same place from a different viewpoint hinges on the allocentric representation provided by the hippocampus and is quite distinct from analogous mental rotation processes applicable to isolated objects (Easton and Sholl, 1995; Wraga et al., 2000; King et al., 2002).

An alternative but compatible theoretical perspective sees the hippocampus as the apex of the ventral stream for visual processing, in which increasingly abstract representations of objects and environment are derived from initially sensory forms (Bussey and Saksida, 2007). Abstraction involves deriving representations which are invariant to low-level sensory change and thus stable over time, providing the basis for recognition memory. For spatial representations of the environment, a key aspect of invariance is view-invariance, since the same location is likely to be encountered from multiple perspectives (Goodale and Milner, 1992).

It is not yet clear exactly how such view-invariant representations are established, but single unit recordings in humans have been broadly consistent with evidence of allocentric spatial firing fields (Ekstrom et al., 2003) and highly invariant responses to images of places as well as non-spatial stimuli (Quiroga et al., 2005). Because of their relatively coarse spatial resolution, it is impossible to investigate neural representations in this level of detail using functional neuroimaging methods. However, recently developed multi-voxel pattern analysis methods show that that different locations in a virtual environment can be distinguished on the basis of the pattern of medial temporal lobe activity they elicit (Hassabis et al., 2009). More generally functional neuroimaging studies have implicated the hippocampus in episodic memory including memory for personally experienced spatial scenes (e.g., Cabeza et al., 2004). Virtual reality methods have been used to investigate navigation, where hippocampal activation is found to correlate with the accuracy with which an individual chooses direct novel routes (Maguire et al., 1998; e.g., Gron et al., 2000; Hartley et al., 2003).

Thus, several lines of theory and convergent experimental evidence implicate the hippocampus in allocentric spatial memory and the ability to represent and manipulate topographical information as distinct from the visual information associated with particular perspective. The current study investigates the possibility that topographical memory could provide a sensitive behavioral index of hippocampal structure and function. Here, topographical memory is assessed through the ability to recognize a place from its spatial layout as distinct from its local visual features. This operational definition leaves open the nature of the processes involved, which may overlap substantially with the allocentric representational role revealed by studies the spatial correlates of firing in hippocampal neurons, and with the hippocampal role in the closely-related theoretical concepts of cognitive maps, (spatial) relational memory, scene-construction, and self-projection reviewed above. We note, for example, that the test demands are closely aligned with Hassabis and Maguire's (2007) definition of the scene-construction process: “the retrieval of relevant semantic and sensory information and, its integration into a coherent spatial context and online maintenance for later manipulation and visualization including possible viewpoint transformation.”

Work with healthy populations suggests that the spatial functions of the hippocampus may be especially sensitive to structural variation. For example, there is evidence that variations in hippocampal volume correlate with navigation expertise (Maguire et al., 2000, 2006) and with the use of spatial strategies (Bohbot et al., 2007). A recent study using diffusion tensor imaging (DTI) showed that performance on a navigation task was associated with greater functional anisotropy (a measure of microstructural integrity) in the hippocampus (Iaria et al., 2008).

Expert navigators (licensed London Taxi Drivers) show structural differences in the hippocampus compared with healthy control subjects (Maguire et al., 2000). Relative to control subjects, they have increased gray matter volume in the posterior hippocampus, and reduced gray matter in the anterior hippocampus. These results have since been replicated in a further study (Maguire et al., 2006) using a larger and better matched control group comprised of bus drivers, who have a very similar role but who follow a small number of fixed routes and are not required to learn or calculate new routes between locations. In both studies, the pattern of anatomical difference is greatest in those with longest experience in the cab, suggesting longitudinal change. Indeed in a further, longitudinal study conducted during the intense 3–4 year training period—“The Knowledge”—that licensed London taxi drivers undergo, Woollett and Maguire (2011) found increased gray matter volume in the posterior hippocampi of successful trainees, but not in those of unsuccessful trainees or matched controls. This result strongly suggests that spatial experience can lead to structural change in the hippocampus.

A further study (Maguire et al., 2003) investigated links between navigation and spatial memory and hippocampal structure in the general (healthy, non-expert) population. A group of 26 right-handed male participants were tested on navigation in a virtual environment previously used in functional neuroimaging studies (Maguire et al., 1998; Burgess et al., 2001). After a minimum of 15 min exploration, subjects were tested on their ability to navigate between identified locations in the virtual town, to draw a map of the town, and to identify scenes from the town (compared with similar scenes in a two alternative forced choice). None of these measures were found to correlate with hippocampal gray matter, despite several steps to relax statistical criteria and avoid the possibility of false negative results. These null findings in the general population suggested that the pattern seen in taxi drivers might reflect the degree or duration of their expertise rather than innate or pre-existing individual differences. However, it is possible that some of the tasks used were insensitive to individual differences in spatial memory. In particular, the environmental scene recognition task showed a rather narrow range of performance, which may have been affected by a ceiling effect. Relatedly, and as with some standardized tests of scene recognition (e.g., the Camden Topographical Recognition Memory Test or CTRMT, Warrington, 1996), the presence of uncontrolled non-spatial cues in target and foil images may have allowed for non-spatial solutions.

To address these and other problems with existing scene recognition paradigms, in previous work (Hartley et al., 2007) we developed a test of topographical memory expressly designed to demand a view-invariant representation of a novel location, and to resist solutions based on alternative strategies. The test used computer-generated landscapes, with viewpoint and global non-spatial properties of the scene (lighting, weather conditions, color, and texture of vegetation) being varied between presentation and testing. We found that patients with damage to the hippocampus were consistently impaired the topographical memory task, which incorporated a brief delay between presentation and testing of each item, but not on a concurrent perceptual matching task or non-spatial tests of memory and perception using the same stimuli. We recently showed (Bird et al., 2010) that performance on the Four Mountains Task could be used to discriminate patients with Mild Cognitive Impairment (MCI) and Alzheimer's Disease (AD) from patients with fronto-temporal dementia. The conditions present with rather similar levels of global cognitive impairment—for example, the groups could not be distinguished using standard tests of cognitive status or memory such as the Mini-Mental States Exam (MMSE) (Folstein et al., 1975), CTRMT (Warrington, 1996), or Doors and People Test (Baddeley et al., 1994). These results combined with our earlier findings suggest that the test is reasonably successful in isolating hippocampal function and thus sensitive to the medial temporal lobe damage which occurs early in the progression of AD but relatively later for patients with other diagnoses. The test may have some application in the assessment of hippocampal function in clinical groups.

Notwithstanding earlier null findings (Maguire et al., 2003), the correlation between navigation expertise and hippocampal structure in taxi-drivers (e.g., Maguire et al., 2000) suggests that variation in hippocampal structure can be linked with spatial competence in non-clinical populations, while very recent genomic findings (Stein et al., 2012) demonstrate that individual differences in structure are subject to experience-independent influences; if the task is sufficiently sensitive and selectively dependent on hippocampal function, we might expect to find a correlation between performance and hippocampal structure in healthy non-experts.

In the current study, we used a variant of the Four Mountains Task to assess topographical memory in healthy adults, and quantify its correlation with measures of gray matter volume. If the Four Mountains Task is a sensitive index of the hippocampus' spatial function, and if variations in hippocampal function are linked to structural variation in the healthy population, then we would expect performance on the task to correlate with measures of gray matter volume within the hippocampus.

Like the studies reviewed above, we use voxel-based morphometry (VBM, described below). VBM is a widely-used general technique for analysis of structural images, which has the advantage of being automated and unbiased (requiring no prespecified region of interest). Importantly VBM can give an indication of where within the hippocampus any correlation is strongest and, equally important, can determine whether any correlation extends beyond the hippocampus (which might indicate a more wide-spread spatial processing network, or alternatively a less selective effect of global organization). Despite these advantages, the VBM processing pipeline offers the potential for gray matter volume estimates for one voxel to be influenced by more distant structures. For this reason we also used a complementary analysis based on automated segmentation of the hippocampus. This approach directly measures the volume of a specified structure, determining its boundaries using local intensity information present in the unsmoothed structural scans. As a byproduct of this analysis we also obtained volume estimates for other, sub-cortical structures.

## Methods

### Participants

Thirty right-handed participants (15 male and 15 female) took part in the study. Ages ranged between 19 and 39 years with a mean of 25.9 years. All participants gave informed consent in accordance with the requirements of the local ethics committee. Volunteers were asked about their health and any reporting relevant neurological or psychiatric history (or other significant health problems) were not recruited to the study.

### Materials

**Figure 1:**
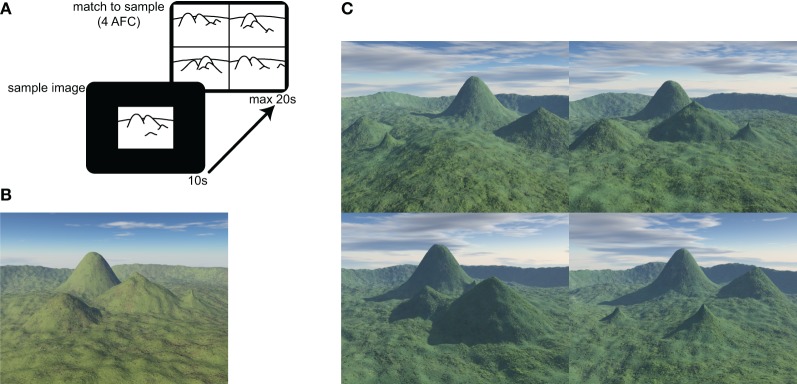
**Four Mountains Task and example stimuli. (A)** The test consists of 30 items. In each item a sample image is presented for 10 s. The four alternative match to sample task is then displayed for up to 20 s. **(B)** Example of sample image. Four distinctive hills are arranged around the camera's focal point, within a semicircular range. **(C)** Example of four alternative forced choice matching task (target shown in **B**). Non-spatial cues (texture, weather, and lighting) and viewpoint are varied between presentation and testing. Lures are generated by systematically varying original topography (see text and Hartley et al., 2007 for further details). The correct response (matching the sample landscape in **B**) for this item is top-right.

The behavioral task was a 30 item, electronic version of the topographical memory component of the Four Mountains Task described in Hartley et al. (2007). The stimuli were all computer-generated landscapes comprised of four hills (of varying shape and size) surrounded by a distant semicircular mountain range (see Figure 1).

Participants were presented with a sample image, which they were required to study for 10 s. They were then immediately presented with four alternative images, one of which (the target image) showed the same topography as the sample image, seen from a novel viewpoint. The participants' task was to identify the target image by pressing a key.

Non-spatial features (lighting, vegetation, weather conditions) of both target and foil landscapes were varied between presentation and testing, such that transient local features of the image could not be relied on to solve the task. The foil images were constructed by systematically varying the topography of the target image (described more fully in Hartley et al., 2007). Briefly, each of the three foils was created using a different method: in one foil the location of one of the four distinctive hills was moved, in another, the locations of two distinctive hills were exchanged, and in the third the shape of one or two of the hills was altered. This approach preserves many local visual and topographical similarities between target and foil landscapes, so that it is difficult to consistently exclude the foils without a representation of the global layout. Combined with the view shift between presentation and testing this means that response strategies based on individual landmarks or local visual features are likely to be ineffective.

### Procedure

#### Behavioral task

Participants read written instructions before attempting four practice items using paper-based stimuli not included in the experimental task. Participants were given feedback on the practice items. Before proceeding to the experimental task, participants were reminded of the instructions: “Your task is to identify which of the four pictures shows the same place as the previous picture. Focus on the layout of the scene (the shape and arrangement of the mountains and other geographical features).” For each item, the target image was displayed for 10 s. The four test images were then displayed for up to 20 s, and participants were instructed to respond “as quickly and accurately as possible” using one of four keys arranged to correspond with the locations of the stimuli, for example pressing the top left key would indicate top left image matched the place in the target image. After each response, the next item was displayed after a 2 s inter-trial interval. The topographical memory score was obtained by counting the number of correct responses out of a maximum of 30 with chance equating to a score of 7.5.

#### MRI

In a separate session, a T1-weighted structural MR image of each participant's brain was acquired, normally in connection with other ongoing research projects. MRI scans were carried out using a GE 3.0T Signa Excite HDx scanner (GE Healthcare) and acquired using a sagittal isotropic 3D FSPGR sequence with the following parameters: Matrix size 256 × 256 × 176, TR 8.03, TE 3.07, Flip angle 20°, 1 mm saggital slices. In plane resolution was 1.13 × 1.13 mm except in seven cases which used a 1.0 × 1.0 mm resolution. We report data from the full sample of 30 participants but have reanalyzed the critical correlations excluding the seven anomalous cases which were identified only after data collection had been completed. These analyses, which do not affect our interpretation of the data, are provided as Supplementary Material.

### VBM preprocessing steps

MRI data were analyzed using standard VBM methods as implemented in SPM5 (Wellcome Department of Cognitive Neurology, London, UK). Each image is pre-processed by reorienting the images to align the anterior commissure in each image. The SPM segmentation algorithm (Ashburner and Friston, 2005) then iteratively combines non-linear registration of the structural image to a standard image (the MNI152 template derived by averaging linearly registered data from 152 healthy participants) with the partitioning of the image into separate maps grey matter, white matter, CSF, and other tissue types, based on the known spatial distribution of each tissue class in the standard brain. The segmentation step also includes a bias correction algorithm which corrects for inhomogeneities in image intensity introduced by various aspects of the MR acquisition process. The resulting images provide an estimate of the density of each tissue type in the participant's brain within each voxel of the standard space. As is now standard (Mechelli et al., 2005b) VBM analyses are based on “modulated” gray matter maps in which the intensity of each voxel is adjusted (using Jacobian determinants derived from the deformation fields used to warp the image into standard space) to reflect the degree to which the corresponding structure has been distorted to fit the template. For example, if a given structure has to be doubled in volume to fit the standard template, the intensities of affected voxels will be halved. Voxels in the modulated gray matter image thus provide a measure of the gray matter volume in the corresponding part of the original image. Finally, the normalized, segmented, modulated tissue maps were smoothed with a 10 mm full-width, half maximum Gaussian kernel. This step is intended to reduce the effects of misregistration and the impact of small individual variations in local anatomy while maintaining sensitivity to reliable, larger-scale volumetric variations.

### Statistical analyses

Local gray matter volume estimates (voxel intensities from the modulated gray matter images) from each individual were entered into a simple linear regression model in SPM5, with score on the topographical memory task as the covariate of interest. T-statistic images were calculated for the strength of the linear association. Voxels containing a low volume of gray matter were excluded from the analysis by using an absolute threshold masking of 0.1. Regions showing a significant correlation (*t* > 3.41, *r* > 0.54, *p* < 0.001, uncorrected) between the VBM gray matter volume estimate and topographical memory were identified and are reported below. The use of an uncorrected threshold is justified by our strong prior hypothesis concerning the hippocampus. Additionally we describe below a complementary analysis based on the entire volume of the hippocampus, which avoids the problem of multiple comparisons inherent in voxel-based analysis.

### Total hippocampal volume

To supplement the VBM analysis, we also analyzed the size of the hippocampus and subcortical structures using mesh-based automated segmentation (FIRST: FMRIB's Integrated Registration and Segmentation Tool). The FIRST algorithm (Patenaude et al., 2011) fits a mesh representing the surface of the hippocampus to the structural image. The model is based on hand-segmented T1 weighted structural images from a 336 participants including healthy adults, children, and patients with hippocampal degeneration. There is a one-to-one correspondence between different individual meshes, and this makes it possible to describe intersubject variation in terms of modes of the model (principle components of mesh deformation) which can be linearly combined to account for the variety of hippocampal shapes and volumes. After linear alignment to a standard space, the model is transformed to the space of the individual participant's brain, and the mesh is fitted by varying the mixture of model components until its vertices are aligned with changes in local signal intensity characteristic of the edge of the hippocampus. This provides a useful measure of total hippocampal volume which can be correlated with behavioral measures. For the statistical analysis of total hippocampal volume we report the correlation of volume with topographical memory score, adopting a threshold of *p* < 0.05 (one-tailed) consistent with our prior hypothesis that the hippocampus will be larger in participants with better topographical memory scores. Other subcortical structures are also automatically segmented as part of the FIRST algorithm—for completeness we report all volume-behavior correlations meeting the same threshold.

## Results

**Figure 2:**
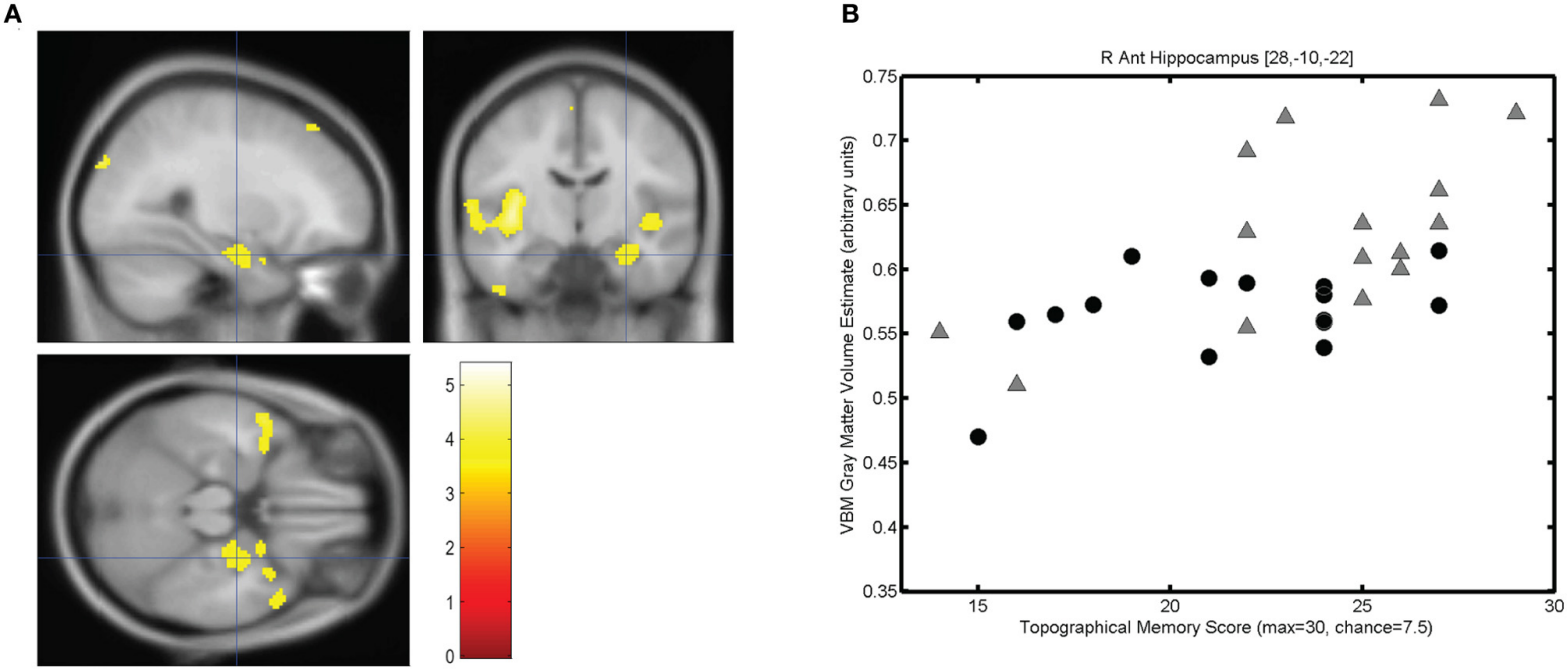
**Correlation between individuals' hippocampal gray matter volume and topographical memory scores. (A)** Regions showing a significant correlation between VBM gray matter volume estimate and score on the 30 item Four Mountains Task. Sagittal, coronal, and axial slices are centered on the peak voxel in the right hippocampus (28, −10, −22). Regions showing a significant correlation (*p* < 0.001, uncorrected) are shown against the canonical MNI152 brain, with the left hemisphere appearing on the left. **(B)** Scattergram showing individual scores and gray matter volume estimates for the peak voxel (*r* = 0.59). Male participants are indicated by gray triangles, female participants by black circles (*r*_♂_ = 0.62, *r*_♀_ = 0.43).

**Figure 3:**
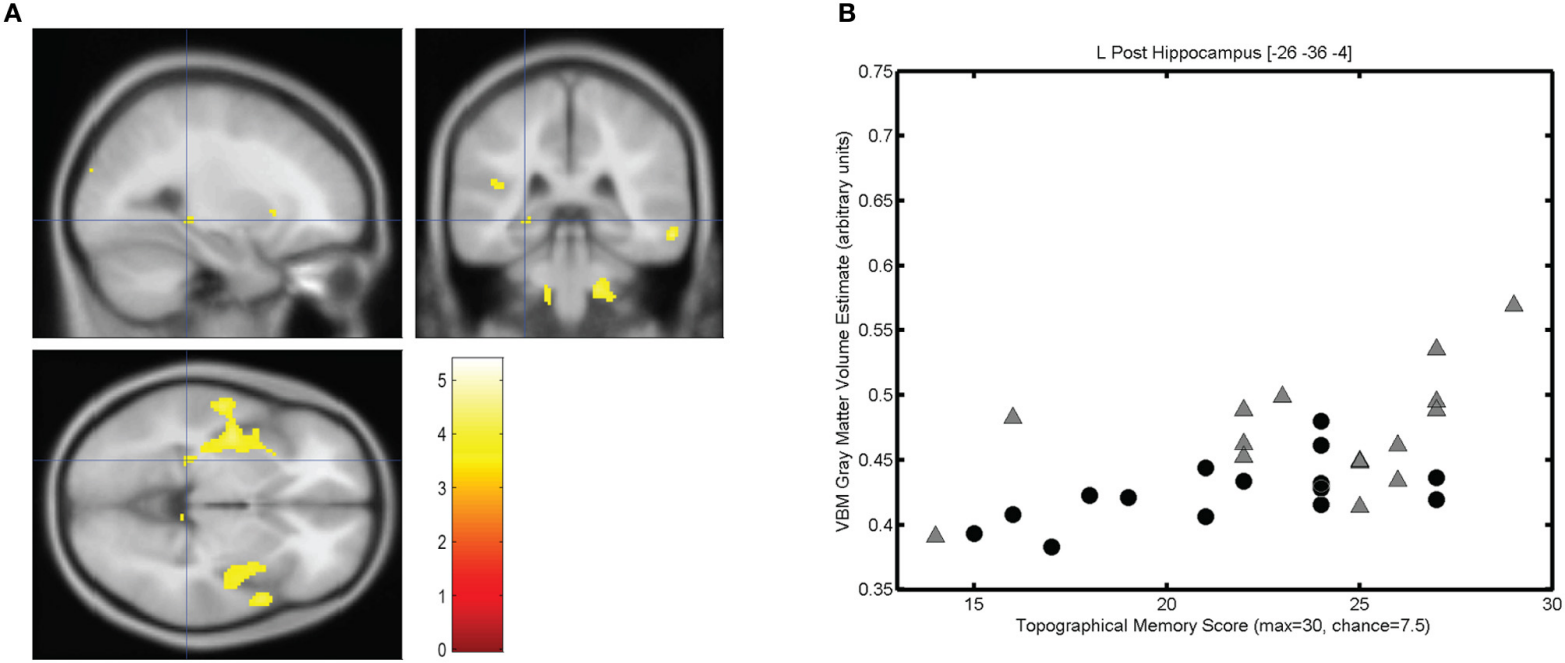
**Correlation between individuals' hippocampal gray matter volume and topographical memory scores. (A)** Regions showing a significant correlation between VBM gray matter volume estimate and score on the 30 item Four Mountains Task. Sagittal, coronal, and axial slices are centered on the peak voxel in the left hippocampus (−26, −36, −4), which is part of a larger cluster encompassing parts of the insula and anterior temporal cortex. Thresholded at *p* < 0.001 (uncorrected). Left hemisphere appears on the left. **(B)** Scattergram showing individual scores and gray matter volume estimates for the peak voxel (*r* = 0.57). Male participants are indicated by gray triangles, female participants by black circles (*r*_♂_ = 0.50, *r*_♀_ = 0.58).

The mean score on the topographical memory test 22.63 (*SD* = 4.06) out of 30. VBM analysis showed that higher topographical memory scores were associated with larger volume estimates in within both hippocampi. Significant positive correlations between volume and topographical memory score were found in voxels in the anterior right hippocampus (Figure 2A) and posterior left hippocampus (Figure 3A). The peak correlation between topographical memory score and volume within the right hippocampus was at 28, −10, −22 (*Z* = 3.44, *r* = 0.59). The peak correlation in the left hippocampus was located at −26, −36, −4 (*Z* = 3.25, *r* = 0.57).

For each participant the VBM volume estimate was extracted for the voxel of peak correlation within left and right hippocampus. Individual memory scores were plotted against the volume of the selected voxel for each participant, illustrating the clear linear association between memory scores and individual gray matter volume estimates for these voxels (see Figures 2B and 3B).

The strongest correlation within the left hippocampus was found at a rather small local peak within a much larger contiguous cluster including insula and superior temporal gyrus. The right hippocampal cluster is focused on the anterior hippocampus but extends into the amygdala and adjacent medial temporal cortex including parts of entorhinal cortex, subiculum, and temporal pole.

Significant correlations between volume and topographical memory score were also found in other regions of the brain. We had no prior hypotheses concerning regions beyond the hippocampus but we report these incidental findings here, since they may indicate that the predicted correlation should not be interpreted as selective. The anatomical locations of these other regions are summarized in Table 1. The most extensive correlations with performance are seen in anterior temporal lobes and insula, whose volumes together account for approximately 75% of the tabulated extra-hippocampal clusters. No voxels were found to be significantly negatively correlated with topographical memory score.

**Table 1:** **Regions showing an association between VBM gray matter volume estimate and performance on the Four Mountains Task**.

**Region**	**L/R**	**Anatomical extent**	**Peak (MNI coordinates)**			**Cluster size (voxels)**	**Z_peak_**
			***x* (mm)**	***y* (mm)**	***z* (mm)**		
	L	Within larger cluster^*^	−26	−36	−4	−	3.25
Hippocampus	R	Hippocampus, amygdala, temporal pole, entorhinal cortex	28	−10	−22	338	3.44
	L	Insula, Rolandic operculum, sup and mid temporal gyri, temporal pole, putamen, hippocampus^*^	−54	8	−14	1730	4.26
Ant temporal/insular cortex (y_peak_ > −25)	R	Insula, Rolandic operculum, sup and mid gyri, temporal pole	52	12	−12	737	4.17
	L	Inf temporal gyrus	−50	−14	−40	60	3.76
	R	Inf temporal gyrus	50	−18	−40	21	3.31
	R	Inf and mid temporal gyri, temporal pole	56	4	−34	26	3.44
	L	Sup temporal gyrus	−52	−30	12	22	3.30
	L	Sup temporal sulus	−44	−36	18	20	3.22
Post-temporal cortex (y_peak_ < −25)	R	Sup temporal sulcus/angular gyrus	46	−68	12	27	3.46
	R	Middle and inferior temporal gyri	60	−40	−12	68	4.42
	L	Sup frontal gyrus	−12	62	34	111	4.16
	R	Sup frontal gyrus	16	58	34	36	3.83
	L	Sup frontal gyrus	−18	66	12	11	3.31
Frontal cortex	L	Sup frontal gyrus/supplementary motor area	−10	0	66	93	3.81
	R	Sup frontal sulcus	30	34	54	14	3.48
	L	Postcentral gyrus/Rolandic operculum	−58	−16	20	137	3.61
Central-cortex	L	Postcentral gyrus	−60	4	10	21	3.32
	L	Precuneus	−12	−68	62	96	4.20
	R	Precuneus	−14	−54	44	25	3.46
Parietal cortex	R	Inf parietal sulcus/angular gyrus	42	−56	44	48	3.98
	R	Sup and mid occipital gyri	26	−90	32	42	3.69
Occitpital cortex	R	Calcarine sulcus	22	−68	16	16	3.25
	midline	–	4	−84	−20	33	3.46
Cerebellum	L	–	−14	−36	−44	18	3.21
	R	–	18	−34	−42	91	3.90

* Left hippocampal peak is part of a larger cluster involving Anterior Temporal and Insular Cortex. Abbreviations: L, Left; R, Right; Sup, Superior; Inf, Inferior; Mid, Middle. *P* < 0.001 uncorrected. Voxel size for VBM analysis is 2 mm × 2 mm × 2 mm. Clusters less than 10 voxels in extent are omitted.

**Figure 4:**
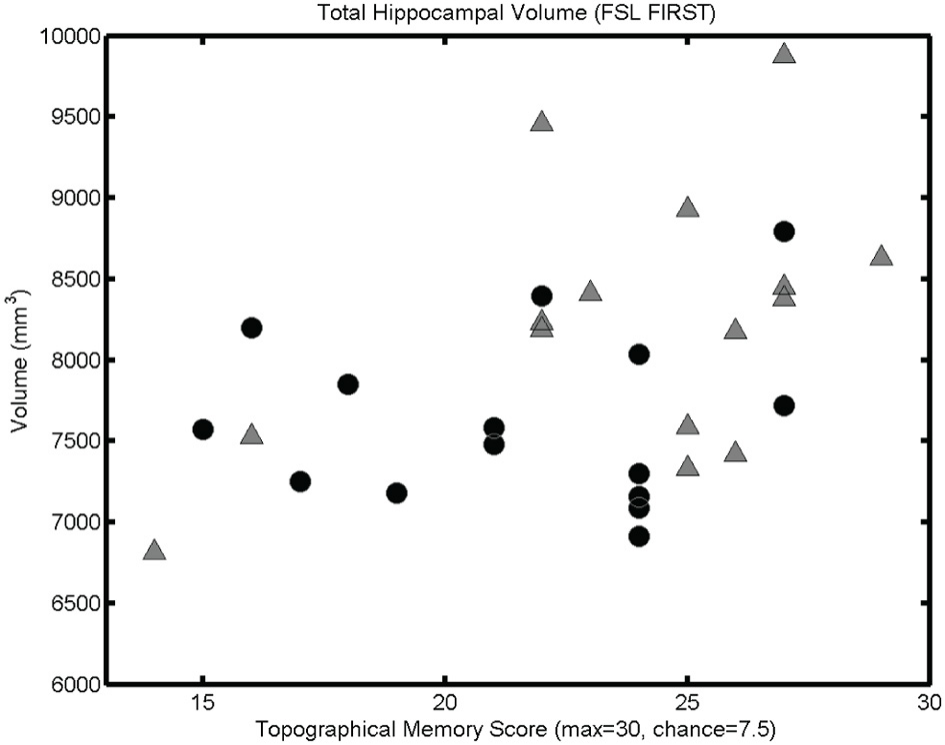
**Mesh-based segmentation results.** A scattergram showing correlation between total hippocampal volume (as determined by automated mesh-based segmentation) and topographical memory scores (*r* = 0.40, *p* < 0.05; *r*_♂_ = 0.48, *p* < 0.05; *r*_♀_ = 0.09, *p* > 0.05). Significant correlations are also found in each of the right and left hippocampi considered independently.

The mesh-based automated segmentation confirmed a statistically significant correlation between total hippocampal volume (summing left and right volumes) and performance on the topographical memory task (*r* = 0.40, *p* < 0.05, see Figure 4, significant correlations *r* > 0.36 are also seen in each hippocampus considered separately).

As with the VBM analysis, mesh-based segmentation of hippocampus and subcortical structures revealed more widespread covariation with volume. Significant positive correlations seen for thalamus (bilaterally *r* > 0.37, *p* < 0.05) and for right amygdala (*r* = 0.43, *p* < 0.05; left amygdala also approaches statistical significance: *r* = 0.31, *p* = 0.051), putamen (bilaterally, *r* > 0.33, *p* < 0.05), and palladium (right only; *r* > 0.37, *p* < 0.05). Notably, the volume of the caudate nucleus was not significantly correlated with performance (left *r* = 0.15, *p* = 0.21 right *r* = 0.22; *p* = 0.12). No area investigated showed a significant negative correlation with topographical memory performance. Indeed, a *post-hoc* mesh-based segmentation of the entire brain (i.e., including white matter and CSF as well as gray matter) using BET2 (FMRIB) showed a significant correlation between total brain volume and performance (*r* = 0.46, *p* < 0.05).

### Effects of sex

Because of the unexpectedly strong relationship between brain size and task performance, we investigated the strength of the observed associations in each sex. Since we had no prior hypothesis concerning sex differences, these incidental observations should be interpreted with caution. In Figures 2B, 3B, and 4, male and female participants are plotted separately (males as triangles, females as circles). Men had significantly larger hippocampi (as assessed by FSL FIRST, *t*_(28)_ = 2.36, *p* < 0.05) and tended to perform better in the topographical memory task (mean♂ = 23.7, median♂ = 25; mean♀ = 21.5, median♀ = 22), however, this difference did not approach statistical significance [*t*_(28)_ = 1.51, *p* = 0.14). Correlation coefficients were calculated independently for male and female participants and VBM gray matter estimates for voxels showing the largest overall correlation with topographical memory—these correlations are of comparable size for each group (e.g., for the right hippocampal peak, *r*_♂_ = 0.62, *r*_♀_ = 0.43) suggesting that structural variation within each sex contributes to the association with topographical memory. This, however, might reflect bias in the selection of the peak voxels (by definition these are voxels where any noise in the data least disrupts the linear association). A more valid indicator of the relative contribution of male and female participants to the observed association is the correlation between total hippocampal volume and performance. Here the correlation appears to be driven largely by the men (*r*_♂_ = 0.48 *p* = 0.07, *r*_♀_ = 0.09 *p* = 0.76).

## Discussion

Our results showed a clear association between hippocampal gray matter volume and healthy individuals' performance on a topographical memory task, in line with our prior hypothesis. Previous work has implicated the hippocampus in allocentric spatial judgment in general (see Burgess, 2008 for review). The current task was previously shown to be selectively impaired in cases of hippocampal damage (Hartley et al., 2007), and to discriminate patients with dementia implicating medial temporal lobe disease from fronto-temporal variants (Bird et al., 2010). The correlational data from the current study do not imply a causal role for structural variation, but this would be a parsimonious explanation for the observed association, in line with previous studies linking hippocampal structure to navigation expertise and spatial strategy (Maguire et al., 2000, 2006; Bohbot et al., 2007; Iaria et al., 2008; Woollett and Maguire, 2011) as well as our earlier work with patients. In this context, it seems likely that structural variations in the healthy hippocampus contribute to individual variation in topographical memory performance.

However, the pattern of volumetric correlations we saw was not restricted to the hippocampus: the association does not appear to be selective. We did not see any region in which local volume estimates were negatively correlated with performance, and indeed we found a correlation between total brain volume and performance. VBM analysis identifies those brain regions that show the strongest correlations, with better performance being specifically associated with the relative enlargement of anterior temporal, insular, and limbic structures including the hippocampus, anterior temporal neocortex, and subcortical structures. These regions included structures (such as the amygdala and superior parts of temporal neocortex) which are not part of the “core” network implicated in spatial cognition and highlighted in studies of scene-construction and self-projection. Because of this the results are not obviously compatible with an interpretation based on selective covariation within a specifically spatial network. Rather we are inclined to speculate that we are seeing the volume variations which result from global factors which determine brain size, influencing its regional organization and the proportion of the available volume given to different structures. Previous studies with much larger sample sizes have indicated that gray matter volumes in these structures covary with one another (amydala, temporal pole, Mechelli et al., 2005a) and are reliably larger in men than in women (amygdala, hippocampus, superior temporal cortex, Good et al., 2001) even when total gray matter volume is taken into account. This suggests that the overall distribution of gray matter in temporal lobes is subject to non-selective global influences which might account for some individual differences in spatial function through their effect on critical brain structures such as the hippocampus. This might include recently identified genetic variations linked specifically to hippocampal volume or more generally to intracranial volume (Stein et al., 2012) it could also include epigenetic effects (such as those underlying sex differences, McCarthy et al., 2009).

The presence of structure-behavior correlations in regions not suspected of spatial function and the fact that our participants did not have any extended training or expertise in topographical memory favor an experience-independent account of our results. Certainly, it is hard to reconcile our results with the idea that correlations between brain structure and performance in the current task result solely from experience-dependent, selective change. There is now strong evidence that such selective structural change is possible (Maguire et al., 2000, 2006; Woollett and Maguire, 2011), but it does not appear to be the whole story. It remains plausible that any initial variation might bias individuals toward particular strategies and experiences leading to further specialization through selective structural change even in non-experts. We cannot separate contributions of innate structural variation and pre-existing experience-dependent change in a cross-sectional study, but studies adopting longitudinal and developmental approaches (cf., Zielinski et al., 2010; Woollett and Maguire, 2011) may shed further light on this question in the future.

One previous study (Maguire et al., 2003) failed to show a correlation between hippocampal structure and behavior in a range of tasks including virtual navigation and scene recognition, despite efforts to relax statistical criteria and thus avoid false negative findings. The current results suggest that the Four Mountains Task may be more sensitive to structural variation than these tasks. One important difference between the Four Mountains Task and the scene recognition test used by Maguire et al. is that the lures are systematically based on the target topography such that only one of the four alternatives offered can be ruled out on the basis of local shape information; discrimination of the target from the other foils requires memory for the layout of the features which must also be insensitive to viewpoint manipulation. In the earlier VBM study and in classic tests of topographical memory (Warrington, 1996) local visual features may be sufficient to identify or exclude scenes.

### Hippocampal contributions to behavior

How might greater hippocampal gray matter volume contribute to performance on the topographical memory task? It is well established from neurophysiological studies in animals that hippocampal neurons encode spatial location in an allocentric form, though the precise form of this code in humans is not yet well understood. One possibility is that individuals with larger hippocampi are able to use the additional neural resources (e.g., neurons, synapses) to rapidly form a richer representation of the layout of the depicted environment, supporting better discrimination of the target scene from the foils. This mechanism might be analogous to that seen in primary visual cortex, where wide variation is seen in the overall size of V1 (Andrews et al., 1997) with visual acuity being correlated with the area of the cortical field (Duncan and Boynton, 2003); by analogy we can think of “topographical acuity” being dependent on the volume of the hippocampus.

Another related possibility is that biological constraints on the ability to form useful allocentric representations drive individual differences in representational strategy. We can distinguish two broad classes of strategy relevant to large scale spatial behavior. One strategy would rely on forming a (hippocampal-dependent) allocentric representation of a location at encoding. This might permit recognition of the same place based on a scene-construction process (Hassabis and Maguire, 2007). An alternative strategy would be to encode local visual features associated with a specific place (independent of their topography). In this view, individuals with larger hippocampi are better able to exploit the allocentric strategy, which is less effective for others. Individuals with smaller hippocampi might come to rely more on local landmark information represented outside the hippocampus. While such a strategy would be useful in everyday life (where there are often unique visual features that serve to distinguish distinct locations), it would be counterproductive in the Four Mountains Task because the same features are deliberately rearranged in the foils to resist visual landmark-based solutions. This strategic account is consistent with an earlier study showing that hippocampal volume correlates with use of an allocentric “place” learning strategy in a task which can also be solved using a non-spatial “response” learning strategy (Bohbot et al., 2007). It may also relate to sex differences in spatial cognition, in which women report greater use of landmark information in spatial tasks (Lawton, 1994; Coluccia and Louse, 2004) and show reduced hippocampal activation (and poorer performance) during virtual navigation (Gron et al., 2000).

In this connection, we noted above that men tend to have larger hippocampi than women (even when total brain volume is taken into account), and that regions showing strong structural-behavioral correlations in the current study overlap with those which are relatively enlarged in men compared with women (Good et al., 2001). We also note that the association between total hippocampal volume and performance on the task is much stronger in men than in women. However, we do not see a significant difference in performance between men and women. Overall, caution is warranted and further investigation is needed to clarify the contribution of sex differences and strategy to the relationship between brain structure and performance on the current task. At this stage it seems possible that sex contributes to global influences on gray matter distribution that are relevant to topographical memory (perhaps favoring one strategy over another), but not to the extent that it reliably leads to performance differences.

### Specificity of the association to spatial function

Our previous studies also employed a non-spatial test using the same class of stimuli (showing in Hartley et al., 2007, that hippocampal damage led to selective impairment of topographical memory). We have not used a non-spatial control condition in the current experiment, so we cannot be certain that the correlation we see in the current study is specific to spatial function. Indeed it is well established that the hippocampus is critical for the formation of new long-term episodic memories, and it would be fairly surprising if volume variation were to affect one aspect of hippocampal memory and not another. However, our previous work (Hartley et al., 2007) suggests that the hippocampus is especially sensitive to spatial information the over short time periods used in the current test, which also make the test more practical to administer (e.g., in clinical settings). If hippocampal volume exerts a more general influence on hippocampal function in healthy people, we might expect this to be evident in sensitive tests of long-term episodic or autobiographical memory, if they can be designed to exclude extra-hippocampal contributions (due to e.g., familiarity, consolidation).

On the other hand even within the domain of spatial memory, other brain structures are believed to make non-spatial contributions to performance on apparently spatial tasks. We would not expect these regions to play a causal role in affecting performance on the current task which is designed to preclude non-spatial strategies. For example, activation of the caudate nucleus is associated with rote-like “response learning” for familiar routes in navigation (Hartley et al., 2003) and with comparable non-spatial strategies in a spatial memory task (Iaria et al., 2003); consistent with this non-topographical contribution to spatial memory and behavior, the volume of the caudate is unrelated to performance on the four mountains task.

### Pattern of local variation within the hippocampus

Although our results were quite clear in showing an overall correlation between hippocampal volume and performance, it would be premature to draw very strong conclusions about the effect on behavior of local structural variation within the hippocampus on the basis of the one study. Our VBM results showed the strongest correlations between hippocampal volume and topographical memory in left posterior- and right anterior-hippocampus, whereas previous studies in expert navigators showed increased volume in the right posterior hippocampus (Woollett and Maguire, 2011) and often *reduced* volume in anterior hippocampi of expert navigators (Maguire et al., 2000, 2006). Further research will be necessary to establish whether the pattern of local variation within the hippocampus is reliable in non-experts, but it should be noted that we would not necessarily expect the parts of the hippocampus which are enlarged in spatially-skilled non-experts to be the same as those which “grow” or “shrink” following training or prolonged expertise with spatial tasks—indeed such structural changes may act to offset or balance the initial state. It is also possible that specific patterns of structural variation might favor different spatial tasks (navigation, topographical memory) perhaps relating to the spatial scale of the underlying representation which varies along the longitudinal axis of the hippocampus (Jung et al., 1994).

### Extra-hippocampal contributions to performance

It remains possible that the extra-hippocampal regions whose volume also covaries with performance could contribute to behavioral variation. We did not have a prior hypothesis concerning these regions, or convergent evidence concerning the effects of extra-hippocampal lesions on the current task, so interpretation of these correlations is necessarily rather speculative. Equally, given that they are of a similar magnitude to the predicted hippocampal association, it would be misleading to overlook the possibility that they play some part in the behavioral variations we see. It is notable that several of the regions whose volume covaried with performance on the Four Mountains Task have previously been implicated in allocentric representation and spatial cognition. For example, an fMRI study of wayfinding (Hartley et al., 2003) found parts of the insula and rhinal cortex were among a small subset of regions, also including the hippocampus, where activity during a wayfinding task predicted individual performance. The discovery of grid cells in the medial entorhinal cortex of the rat has clearly implicated cortical areas downstream of the hippocampus in representing allocentric spatial information (Hafting et al., 2005). A recent fMRI study (Doeller et al., 2010) identified a region within human entorhinal cortex which shows the characteristic six-fold allocentric orientation tuning expected of a grid cell population. The same study also found neural adaptation to movement direction suggestive of grid cell populations in lateral temporal cortex. As these anterior temporal regions are directly implicated in the representation of large scale space, we cannot rule out the possibility that structural variations within them could play a causal role in the observed association with topographical memory.

### Potential clinical applications

Any measure of spatial function sensitive to structural variation in the general population could prove useful in assessing clinical conditions. Structural change in the hippocampus is associated with diagnostic status in AD and MCI (an intermediate diagnosis which often precedes progression to AD), with 6% annual volume loss in AD and 3% in MCI compared with less than 1% in normal ageing (Morra et al., 2009). These volumetric variations correlate only weakly with global measures cognitive function such as MMSE (Folstein et al., 1975). To be of practical value, more selective measures are needed to directly target hippocampal function, while remaining capable of being administered straightforwardly and quickly in the clinic without specialist equipment. The current results suggest that a brief test of topographical memory may fulfill these criteria and indeed preliminary work with the Four Mountains test shows promise in identifying patients with specifically hippocampal pathology. For example, Bird et al. (2010) showed that patients with temporal variants of fronto-temporal dementia (where early anterior, rather than medial, temporal atrophy is expected) show no clear impairment on the mountains task, whereas patients with AD, amnestic MCI and focal hippocampal damage do (Hartley et al., 2007; Bird et al., 2010; Pengas et al., 2010).

## Conclusion

We found clear evidence that hippocampal volume in healthy individuals is correlated with performance on a topographical memory task previously found to be selectively impaired in hippocampal patients. However, the effect did not seem to be driven by *selective* variation in the hippocampus—we also noted more widespread correlations between topographical memory performance, and an association with total brain volume. Outside the hippocampus, the most extensive correlations were concentrated around the anterior temporal neocortex, nearby subcortical structures and insula. Taken together with earlier findings the results suggest that global variations in brain morphology affect the volume of the hippocampus and its specific contribution to topographical memory. The behavioral effects might arise through the direct impact of resource constraints on “topographical acuity”, and perhaps indirectly by encouraging the use of non-allocentric strategies which rely on extra-hippocampal representations.

### Conflict of interest statement

The authors declare that the research was conducted in the absence of any commercial or financial relationships that could be construed as a potential conflict of interest.
